# Temporal profiling of changes in phosphatidylinositol 4,5-bisphosphate, inositol 1,4,5-trisphosphate and diacylglycerol allows comprehensive analysis of phospholipase C-initiated signalling in single neurons^1^

**DOI:** 10.1111/j.1471-4159.2008.05587.x

**Published:** 2008-11

**Authors:** Carl P Nelson, Stefan R Nahorski, R A John Challiss

**Affiliations:** Department of Cell Physiology & Pharmacology, University of LeicesterLeicester, United Kingdom

**Keywords:** phospholipase C, phosphatidylinositol 4,5-bisphosphate, Tubby protein, SH-SY5Y, hippocampal neuron

## Abstract

Phosphatidylinositol 4,5-bisphosphate (PIP_2_) fulfils vital signalling roles in an array of cellular processes, yet until recently it has not been possible selectively to visualize real-time changes in PIP_2_ levels within living cells. Green fluorescent protein (GFP)-labelled Tubby protein (GFP-Tubby) enriches to the plasma membrane at rest and translocates to the cytosol following activation of endogenous Gα_q/11_-coupled muscarinic acetylcholine receptors in both SH-SY5Y human neuroblastoma cells and primary rat hippocampal neurons. GFP-Tubby translocation is independent of changes in cytosolic inositol 1,4,5-trisphosphate and instead reports dynamic changes in levels of plasma membrane PIP_2_. In contrast, enhanced GFP (eGFP)-tagged pleckstrin homology domain of phospholipase C (PLCδ1) (eGFP-PH) translocation reports increases in cytosolic inositol 1,4,5-trisphosphate. Comparison of GFP-Tubby, eGFP-PH and the eGFP-tagged C1_2_ domain of protein kinase C-γ [eGFP-C1(2); to detect diacylglycerol] allowed a selective and comprehensive analysis of PLC-initiated signalling in living cells. Manipulating intracellular Ca^2+^ concentrations in the nanomolar range established that GFP-Tubby responses to a muscarinic agonist were sensitive to intracellular Ca^2+^ up to 100–200 nM in SH-SY5Y cells, demonstrating the exquisite sensitivity of agonist-mediated PLC activity within the range of physiological resting Ca^2+^ concentrations. We have also exploited GFP-Tubby selectively to visualize, for the first time, real-time changes in PIP_2_ in hippocampal neurons.

Phosphatidylinositol 4,5-bisphosphate (PIP_2_) is the primary substrate for cellular phospholipase C (PLC) and phosphoinositide 3-kinase (PI3-kinase) activities, generating the second messengers inositol 1,4,5-trisphosphate (IP_3_) and diacylglycerol (DAG) and phosphatidylinositol 3,4,5-trisphosphate respectively (Berridge 1993; Toker and Cantley 1997). As well as being a key substrate for second messenger-generating enzymes and providing a target for the membrane-association of a variety of protein domains, PIP_2_ is also an important signalling molecule in its own right and is implicated in membrane trafficking, actin cytoskeleton remodelling and the regulation of ion channels and transporters (Cremona and De Camilli 2001; McLaughlin and Murray 2005; Suh and Hille 2005; Ling *et al.* 2006). For example, in the CNS a number of Gα_q/11_-coupled receptors modulate membrane excitability by inhibiting the KCNQ2/3 current (see Delmas and Brown 2005) via distinct receptor-dependent mechanisms. Angiotensin and muscarinic acetylcholine (mACh) receptors inhibit KCNQ2/3 channels by depleting PIP_2_ (Suh and Hille 2002; Zaika *et al.* 2006), whereas activation of Gα_q/11_-coupled bradykinin and ATP receptors suppress current through IP_3_- and Ca^2+^/calmodulin-dependent, PIP_2_-independent mechanisms (Gamper and Shapiro 2003; Zaika *et al.* 2007). As both the substrate and products of PLC can independently influence neuronal activity via distinct mechanisms, the ability selectively to visualize real-time changes in PIP_2_, IP_3_ and DAG is highly desirable for the further study of PLC signalling *in vivo*.

The development of the enhanced green fluorescent protein (eGFP)-labelled pleckstrin homology domain of PLCδ1 (eGFP-PH; Stauffer *et al.* 1998; Varnai and Balla 1998) has provided a means of visualizing real-time changes in PLC activity, by exploiting the high affinity and selectivity of this eGFP-PH domain for PIP_2_ (Hirose *et al.* 1999). The eGFP-PH probe enriches to the plasma membrane and on activation of PLC, translocates to the cytosol (Stauffer *et al.* 1998; Varnai and Balla 1998). The relative causal contributions of PIP_2_ depletion at the plasma membrane and elevation of IP_3_ in the cytoplasm to eGFP-PH translocation have been widely debated (see Varnai and Balla 2006). A number of studies have suggested a predominant role for changes in PIP_2_ in the dynamics of eGFP-PH translocation (Varnai and Balla 1998; van der Wal *et al.* 2001; Winks *et al.* 2005), however, the eGFP-PH domain of PLCδ1 exhibits (at least *in vitro*) a higher affinity for IP_3_ than for PIP_2_ (Hirose *et al.* 1999), and theoretical (Xu *et al.* 2003) and empirical evidence (Hirose *et al.* 1999; Okubo *et al.* 2001; Nash *et al.* 2002, 2004) has accrued indicating that eGFP-PH translocation in live cells may primarily reflect changes in cytosolic IP_3_. Clearly, these data indicate that eGFP-PH does not represent a truly selective tool for the study of dynamic changes in PIP_2_ levels in cells and a more PIP_2_-selective biosensor is needed.

The observation that Tubby protein is localized to the plasma membrane via a novel PIP_2_-binding domain (Santagata *et al.* 2001) raises the possibility that this might be an alternative candidate for a PIP_2_ biosensor. A GFP-labelled version of the full-length Tubby protein was found to enrich to the plasma membrane when recombinantly expressed in a variety of cell backgrounds (Santagata *et al.* 2001). Intriguingly, on activation of Gα_q/11_-coupled receptors GFP-Tubby rapidly translocated from membrane to cytosol and ultimately (within 2 h) to the nucleus, where it has been proposed to act as a transcriptional regulator (Boggon *et al.* 1999; Santagata *et al.* 2001). Recent reports describe the use of a fluorescently labelled, modified form of the *C*-terminal domain (amino acids 248–505) of Tubby [R332H-Tubby (248–505)-yellow fluorescent protein] to visualize changes in PIP_2_ levels in human embryonic kidney 293 cells (Quinn *et al.* 2008) and to assess bradykinin-stimulated PIP_2_ synthesis in sympathetic neurons (Hughes *et al.* 2007), suggesting that probes based on the Tubby protein might provide specific biosensors for PIP_2_.

Therefore, we set out to investigate further the acute translocation of (full-length) GFP-Tubby on Gα_q/11_-coupled receptor activation to establish whether this can be utilized as an index of real-time changes in plasma membrane PIP_2_ levels in live cells. Initially, we investigated the translocation of GFP-Tubby, in comparison with eGFP-PH and the established biosensor for DAG [eGFP-C1(2); Oancea *et al.* 1998] in SH-SY5Y human neuroblastoma cells. We have extensive quantitative knowledge of phosphoinositide turnover in these cells from earlier studies from our laboratory (e.g. Willars *et al.* 1998), making them an ideal model system in which to assess a potential PIP_2_ biosensor. We have established that GFP-Tubby translocation on PLC activation reflects dynamic changes in plasma membrane PIP_2_, in both neuroblastoma cells and cultured rat hippocampal neurons. In contrast, the translocation of eGFP-PH predominantly reports changes in cytosolic IP_3_, at least in the cell systems investigated in this study. GFP-Tubby is therefore a potential real-time fluorescent biosensor, suitable for the visualization of changes in PIP_2_ levels in live cells and we have used it here to evaluate the Ca^2+^-sensitivity of agonist-mediated PLC activity in SH-SY5Y cells.

## Materials and methods

### Materials

Cell culture supplies and lipofection reagents were obtained from Invitrogen (Paisley, UK). Thermolysin, pronase, Dnase I, poly-d-lysine, cytosine arabinoside and methacholine (MCh) were provided by Sigma-Aldrich (Poole, UK). Tocris Bioscience (Bristol, UK) supplied wortmannin (Wort) and LY294002, while Fluo-4 AM and Fura-Red-AM were obtained from Molecular Probes (Leiden, The Netherlands).

### Neuroblastoma cell culture and transfections

SH-SY5Y cells were maintained in minimum essential medium supplemented with 100 U/mL penicillin, 100 μg/mL streptomycin, 2.5 μg/mL amphotericin B, 2 mM l-glutamine and 10% newborn calf serum. Cells were maintained at 37°C in a humidified atmosphere of O_2_/CO_2_ (19 : 1) and were routinely split 1 : 5 every 3–4 days, using trypsin–EDTA. For experiments, cells were seeded onto 25 mm glass coverslips for 24 h prior to transient transfection (where appropriate) with 0.5 μg of eGFP-PH, full-length GFP-Tubby or eGFP-C1(2) plasmid DNA, using Lipofectamine2000 (1 : 3 ratio). For co-expression experiments, cells were transfected as above, but with 1.0 μg of dsRed-IP_3_ 3-kinase plasmid DNA in addition to 0.5 μg of GFP-labelled biosensor plasmid DNA. Experiments were performed 24–48 h post-transfection.

### Hippocampal neuron culture and transfection

Hippocampal neurons were prepared from 1-day-old Lister Hooded rats as described previously (Nash *et al.* 2004; Willets *et al.* 2004). Briefly, isolated hippocampi were chopped and treated with pronase E (0.5 mg/mL) and thermolysin (0.5 mg/mL) in a HEPES-buffered salt solution [Hank’s balanced salt solution (in mM): NaCl 130, HEPES 10, KCl 5.4, MgSO_4_ 1.0, glucose 25 and CaCl_2_ 1.8, pH 7.2) for 30 min. The tissue was re-suspended in Hank’s balanced salt solution supplemented with 40 μg/mL Dnase I and triturated through a fire-polished glass pipette. Following centrifugation (400 *g*, 3 min) and further trituration, cells were re-suspended in Neurobasal medium containing B27 supplement, 10% heat-inactivated foetal calf serum, penicillin (100 U/mL), streptomycin (100 μg/mL), l-glutamine (0.5 mM), sodium pyruvate (1 mM) and l-serine (1 mM) and plated onto poly-d-lysine-coated 25 mm glass coverslips. After 24 h, cytosine arabinoside (5 μM) was added to inhibit glial cell proliferation and a further 48 h later, cells were transferred to serum-free Neurobasal medium. Cells were transfected after 11 days *in vitro* (DIV) using Lipofectamine2000, as described above. Neurons were routinely imaged at 12–15 DIV.

### Calcium imaging

Cells were loaded with Fluo-4 AM (2 μM, 40–60 min) before mounting on the stage of an Olympus IX70 inverted epifluorescence microscope. Cells were incubated at 37°C using a temperature controller and microincubator (PDMI-2 and TC202A; Burleigh, Harpenden, UK) and were continuously perfused with Krebs–Henseleit buffer (composition in mM: NaCl 118, KCl 4.7, CaCl_2_ 1.3, KH_2_PO_4_ 1.2, MgSO_4_ 1.2, Na HCO_3_ 25, HEPES 5 and glucose 10). Cells were imaged at a rate of 1–2 Hz using an Olympus FV500 confocal microscope (Olympus Europa, Hamburg, Germany) fitted with a 60× oil immersion objective lens. Fluo-4 was excited using the 488 nm line of an argon ion laser, and emissions over 505 nm were collected. Increases in intracellular Ca^2+^ were defined as *F*/*F*_0_ where *F* was the fluorescence at any given time, and *F*_0_ was the initial basal level of Ca^2+^.

### Confocal imaging of fluorescent biosensors

Cells expressing GFP-labelled biosensors [eGFP-PH, GFP-Tubby and eGFP-C1(2)] were imaged using an Olympus FV500 laser scanning confocal IX70 inverted microscope and continuously perfused with Krebs–Henseleit buffer at 37°C. Cells were excited via the 488 nm line of the argon laser and GFP emissions were collected at 505–560 nm. Increases in signal were calculated as the *F*/*F*_0_ increase in cytosolic GFP levels. In co-imaging experiments, GFP and dsRed were sequentially excited via the 488 nm line of the argon laser and a 543 nm helium–neon laser respectively. GFP and dsRed emissions were collected at 505–560 and > 660 nm respectively. In experiments requiring the co-imaging of Ca^2+^ and GFP-labelled biosensors, transfected cells were loaded with Fura-Red (3 μM, 1 h) and excited by the 488 nm line of an argon ion laser. Emissions from GFP-labelled biosensors and Fura-Red were collected at 505–560 and > 660 nm respectively.

### Data analysis and statistics

Data were analysed using graphpad Prism 4.0 (San Diego, CA, USA). Data were presented throughout as mean ± SEM from three or more coverslips and statistical comparisons were made using Student’s unpaired *t*-test or one way anova followed by Bonferroni’s or Dunnett’s post-test (statistical significance is indicated as *p<0.05, **p<0.01 or ***p<0.001, throughout).

## Results

### Expressing GFP-Tubby, eGFP-PH and eGFP-C1(2) in SH-SY5Y cells

**Fig. 1:**
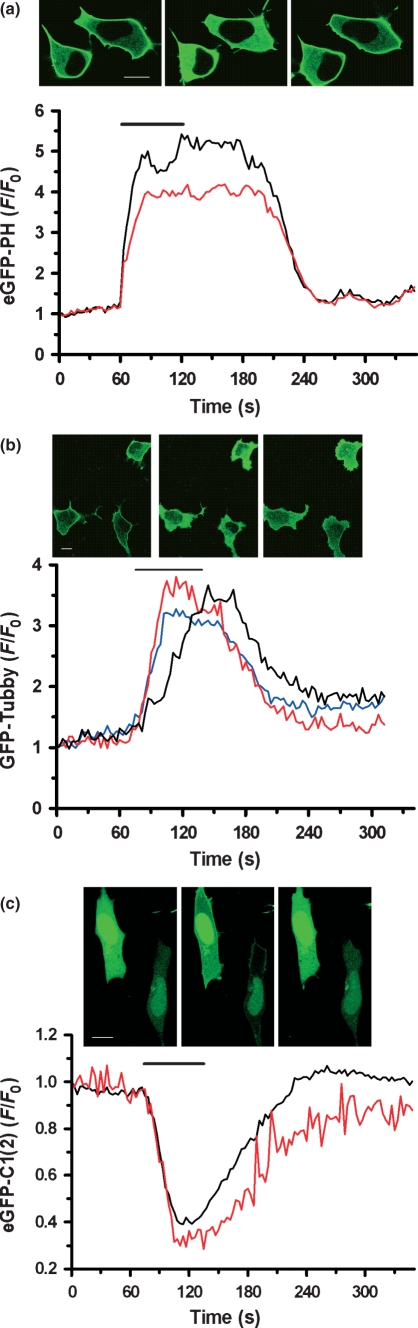
Translocation of GFP-labelled biosensors in response to mACh receptor activation in SH-SY5Y cells. Representative traces and confocal images of SH-SY5Y cells expressing eGFP-PH (a), GFP-Tubby (b) or eGFP-C1(2) (c) in response to MCh (100 μM; black horizontal bars). Data were expressed as a ratio change in cytosolic fluorescence emission (*F*) relative to the initial basal fluorescence (*F*_0_). Data were represented as (*F*/*F*_0_) − 1 (for eGFP-PH and GFP-Tubby) or 1 − (*F*/*F*_0_) [for eGFP-C1(2)]. Scale bars, 10 μm.

To investigate the potential of the GFP-Tubby construct to act as a single cell fluorescent biosensor for PIP_2_, we transiently expressed GFP-Tubby (Fig. 1b) [and for comparison, eGFP-PH (Fig. 1a) and eGFP-C1(2) (Fig. 1c)] in SH-SY5Y neuroblastoma cells. Under basal conditions, eGFP-PH predominantly localized to the plasma membrane, with a lower level of fluorescence in the cytoplasm, as has been reported previously in SH-SY5Y cells (Nash *et al.* 2001). GFP-Tubby exhibited a similar distribution to eGFP-PH, with the exception of a significant nuclear localization of this probe in some cells, as has been observed in other cell-types (Santagata *et al.* 2001). Consistent with previous reports (Oancea and Meyer 1998; Bartlett *et al.* 2005) eGFP-C1(2) fluorescence was observed in both nuclear and cytoplasmic compartments under basal conditions.

On stimulation of the endogenous M_3_ mACh receptor population expressed in SH-SY5Y cells (Lambert *et al.* 1989) with MCh (100 μM), a robust translocation of eGFP-PH and GFP-Tubby from plasma membrane to cytosol was observed (Fig. 1a and b). No obvious change in nuclear localization of GFP-Tubby was observed over the time-course of our experiments (data not shown). Stimulation of SH-SY5Y cells expressing eGFP-C1(2) with MCh (100 μM) elicited a decrease in cytosolic fluorescence (Fig. 1c). Translocation of all three probes to MCh occurred in a concentration-dependent manner, allowing concentration–response curves to be constructed (see Fig. S1) and pEC_50_ values to be determined (Table S1). Mean pEC_50_ estimates for MCh-mediated translocation of eGFP-PH (5.19 ± 0.11) and eGFP-C1(2) (4.89 ± 0.23) were not significantly different to one another, but MCh was significantly less potent in translocating GFP-Tubby (pEC_50_ = 4.53 ± 0.21) than eGFP-PH (*p*<0.05; Table S1). Comparison of the time-courses of translocation of each probe revealed that eGFP-C1(2) translocated most rapidly, with a mean *t*_10–90_ of 31 s, significantly faster than that of eGFP-PH (*p*<0.05; Table S1). GFP-Tubby translocation occurred substantially slower (*p*<0.001) than either of the other two probes, with a mean t_10–90_ of 59 s (Table S1).

One limitation of the use of protein domains with high affinity for inositol phospholipids (such as the PH domain of PLCδ1) is that their over-expression might bind to and thereby preclude hydrolysis (in this case, of PIP_2_), as has previously been suggested for eGFP-PH (Varnai and Balla 1998). We therefore investigated intracellular Ca^2+^ release in response to MCh (using Fura-Red as a Ca^2+^ indicator) in cells expressing each of the GFP-labelled biosensors, compared with untransfected control cells. Ca^2+^ responses to 1 μM MCh (given as 1 − *F*/*F*_0_ self-ratios) were significantly lower in cells expressing eGFP-PH (0.26 ± 0.02; 20 cells from three coverslips) compared with untransfected control cells (0.32 ± 0.01; 27 cells from three coverslips) (*P*<0.01). In contrast, neither GFP-Tubby [0.33 ± 0.03 (+ GFP−Tubby) vs. 0.39 ± 0.02 (control)] nor eGFP-C1(2) [0.32 ± 0.02 (+ eGFP−C1(2)) vs. 0.32 ± 0.03 (control)] significantly affected agonist-induced intracellular Ca^2+^ responses in SH-SY5Y cells. These data indicate that expression of eGFP-PH, but not GFP-Tubby, can attenuate agonist-mediated PLC activity.

### The effect of over-expression of dsRed2-IP_3_ 3-kinase on intracellular Ca^2+^ release and translocation of eGFP-PH, eGFP-C1(2) and GFP-Tubby

**Fig. 2:**
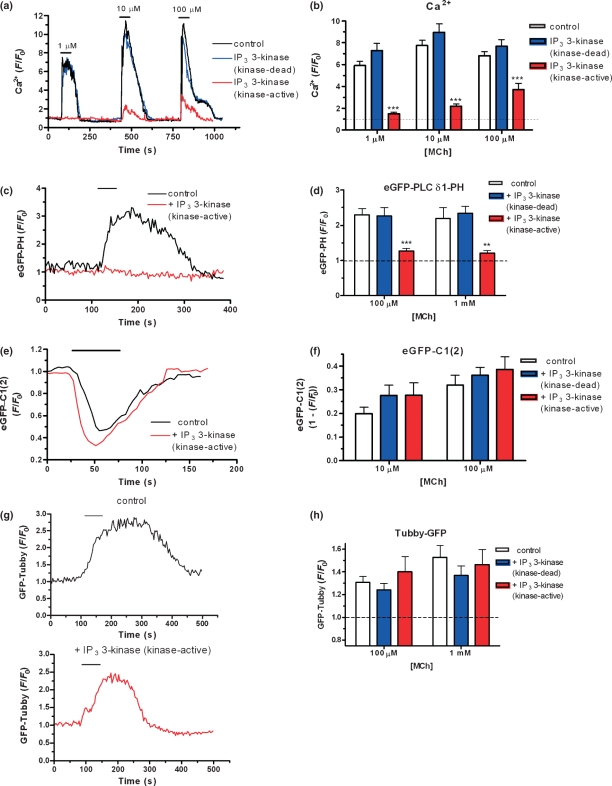
IP_3_ 3-kinase over-expression attenuates Ca^2+^ and eGFP-PH, but not eGFP-C1(2) or GFP-Tubby, responses to MCh in SH-SY5Y cells. (a) Representative traces illustrating changes in intracellular Ca^2+^ in SH-SY5Y cells in response to MCh (indicated by black horizontal bars), as measured by increases in the fluorescence of Fluo-4 AM. Data were expressed as a ratio change in cytosolic fluorescence emission (*F*) relative to the initial basal fluorescence (*F*_0_). Data were shown for un-transfected cells (control; black trace), cells expressing dsRed2-IP_3_ 3-kinase (kinase-dead; blue trace), and cells expressing dsRed2-IP_3_ 3-kinase (kinase-active; red trace). (b) Cumulative data showing increases in intracellular Ca^2+^ in SH-SY5Y cells in response to MCh for untransfected cells (control; open bars), cells expressing dsRed2-IP_3_ 3-kinase (kinase-dead; blue shaded bars), and cells expressing dsRed2-IP_3_ 3-kinase (kinase-active; red shaded bars). Data were expressed as mean ± SEM for 12–104 cells from at least three separate coverslips. Representative traces of eGFP-PH (c), eGFP-C1(2) (e) and GFP-Tubby (g) translocation in response to MCh (100 μM; black horizontal bar) in SH-SY5Y cells expressing biosensor alone (black trace) or co-expressed with dsRed2-IP_3_ 3-kinase (kinase-active) (red trace). Cumulative data representing eGFP-PH (d), eGFP-C1(2) (f) and GFP-Tubby (h) translocation in response to MCh (10 μM–1 mM, as indicated) in SH-SY5Y cells expressing biosensor alone (open bars), or co-expressing biosensor with either dsRed2-IP_3_ 3-kinase (kinase-dead; blue shaded bars), or dsRed2-IP_3_ 3-kinase (kinase-active; red shaded bars). Data were expressed as mean ± SEM for five or more cells from at least three separate coverslips. Differences between cell populations were determined by one-way anova and Dunnett’s *post hoc* test (**p<0.01; ***p<0.001). Scale bars, 10 μm.

To investigate the role of IP_3_ production in the translocation of eGFP-PH and GFP-Tubby, we have examined the effect of over-expressing dsRed2-IP_3_ 3-kinase on the translocation profile of each probe, as we have previously performed for eGFP-PH in other cell systems (Nash *et al.* 2002, 2004). Over-expression of the ‘kinase-active’ dsRed2-IP_3_ 3-kinase significantly reduced the Ca^2+^ response to 1, 10 and 100 μM MCh, while expression of a ‘kinase-dead’ mutant of dsRed2-IP_3_ 3-kinase had no effect (Fig. 2a and b), indicating an enhanced metabolism of IP_3_ in the subpopulation of cells over-expressing the ‘kinase-active’ 3-kinase construct.

Figure 2c illustrates a representative trace from a pair of SH-SY5Y cells, each expressing eGFP-PH, but only one co-expressing dsRed2-IP_3_ 3-kinase. On addition of MCh (100 μM), the cell expressing eGFP-PH alone exhibited a robust elevation in cytosolic fluorescence (Fig. 2c). In contrast, in the cell co-expressing eGFP-PH and dsRed2-IP_3_ 3-kinase, no response was observed. Similar results were obtained in a number of experiments using both 100 μM and 1 mM MCh, with highly significantly lower responses observed in cells expressing dsRed2-IP_3_ 3-kinase than in cells expressing either eGFP-PH alone or eGFP-PH in combination with the ‘kinase-dead’ 3-kinase construct (summarized in Fig. 2d). In contrast, co-expression of dsRed2-IP_3_ 3-kinase with eGFP-C1(2) had no influence on the translocation of the DAG sensor to either 10 or 100 μM MCh (Fig. 2a and b), suggesting that the loss of Ca^2+^-mediated positive feedback on to PLC does not account for the reduced translocation of eGFP-PH on co-expression of dsRed2-IP_3_ 3-kinase. These data therefore suggest that in SH-SY5Y cells, eGFP-PH translocation in response to mACh receptor stimulation is largely dependent on IP_3_ generation. In addition, GFP-Tubby responses to MCh (100 μM or 1 mM) were similar in SH-SY5Y cells regardless of whether the probe was expressed alone or co-expressed with either ‘kinase-active’ or ‘kinase-dead’ dsRed2-IP_3_ 3-kinase (Fig. 2c and d). Translocation of GFP-Tubby in response to mACh receptor stimulation therefore does *not* reflect dynamic changes in cytosolic IP_3_.

### GFP-Tubby translocation reports dynamic changes in PIP_2_

**Fig. 3:**
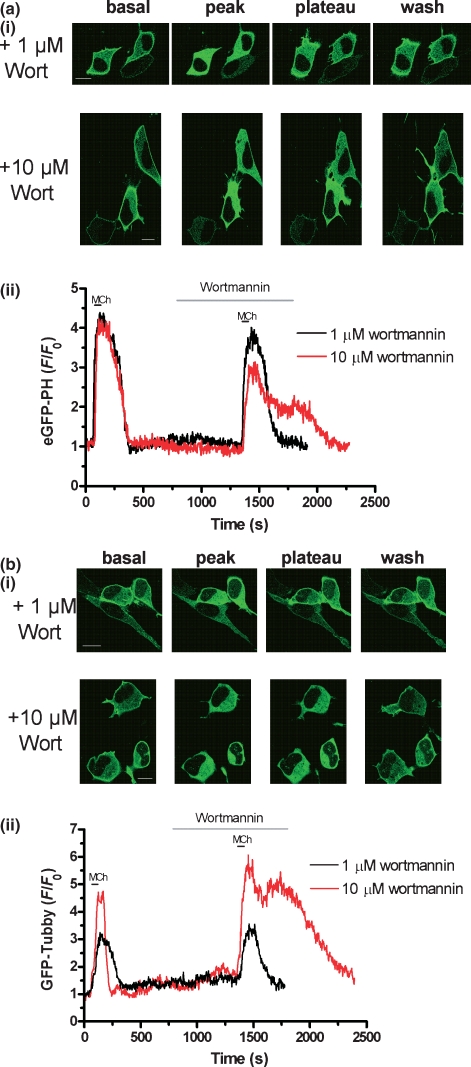
The effect of PI4-kinase inhibition on translocation of GFP-labelled biosensors in response to MCh in SH-SY5Y cells. Representative images (i) and traces (ii) of eGFP-PH (a) and GFP-Tubby (b) translocation in response to MCh (1 mM; black horizontal bar) in cells in the presence of pre-incubated 1 μM (black trace) or 10 μM (red trace) wortmannin (as indicated by grey horizontal bar). Data were expressed as a ratio change in cytosolic fluorescence emission (*F*) relative to the initial basal fluorescence (*F*_0_) and are representative of at least eight cells from three or more coverslips. Scale bars, 10 μm.

To investigate the extent to which eGFP-PH and GFP-Tubby translocation in response to MCh in SH-SY5Y cells depends on dynamic changes in plasma membrane PIP_2_, we investigated the effect of PI4-kinase inhibition on the temporal profile of translocation of these probes. Cells were stimulated initially with MCh (1 mM) alone and then again following incubation with Wort, either at 1 μM (which should fully inhibit PI3-kinase activity, but not PI4-kinases; Balla 2001), or 10 μM (a concentration known to inhibit both PI3- and 4-kinase activities; Nakanishi *et al.* 1995; Willars *et al.* 1998). Wort treatment alone had no significant effect on either eGFP-PH or GFP-Tubby localization (data not shown), but peak eGFP-PH responses in the presence of 10 μM (but not 1 μM) Wort were significantly lower (by ∼22%) than in cells not exposed to Wort (Fig. S3a and Table S2), consistent with an attenuated IP_3_ response in the presence of Wort (Willars *et al.* 1998). The most striking difference was that in the presence of 10 μM (but not 1 μM) Wort, the eGFP-PH response to 1 mM MCh was maintained at a plateau level (44 ± 6% of peak response remaining 240 s after peak) for as long as Wort was perfused on to the cells (Fig. 3a and Table S2). On washout of Wort the response fully returned to baseline levels [Fig. 3a(ii)], suggesting that PI4-kinase activity is necessary for complete re-association of eGFP-PH with the plasma membrane, consistent with its membrane localization being because of an interaction with PIP_2_.

Similar experiments were performed on SH-SY5Y cells expressing GFP-Tubby (Fig. 3b). Pre-incubation with 1 μM Wort had no effect on GFP-Tubby translocation in response to MCh (1 mM). However, in the presence of 10 μM Wort, peak GFP-Tubby responses were significantly enhanced (by ∼66%) in comparison with the same cells prior to Wort treatment (Fig. S3b and Table S2). Similar to eGFP-PH, GFP-Tubby responses to MCh were maintained at a plateau level in the presence of 10 μM (but not 1 μM) Wort, until washout of Wort when the response returned to baseline levels (Fig. S3b and Table S2). However, a more substantial proportion of the peak GFP-Tubby response was maintained in the presence of 10 μM Wort (79 ± 6% of peak response remaining 240 s after peak), suggesting that PI4-kinase activity is essential for the re-localization of GFP-Tubby to the plasma membrane. These data therefore suggest that PIP_2_ re-synthesis is essential for the re-association of GFP-Tubby with the plasma membrane.

Similar data were obtained using the PI kinase inhibitor LY294002, which at high concentrations has been reported to inhibit PI4-kinase activity (Downing *et al.* 1996; Willars *et al.* 1998). These data are summarized in Table S2. Overall, LY294002 had qualitatively similar effects on eGFP-PH and GFP-Tubby translocation in response to MCh as 10 μM Wort, providing strong support for the notion that the activity of PI4-kinases is essential for the re-localization of GFP-Tubby and (to a lesser extent) eGFP-PH following agonist removal.

### GFP-tagged biosensors can be used to determine the Ca^2+^-dependency of PLC activation by Gα_q/11_-coupled receptors

**Fig. 4:**
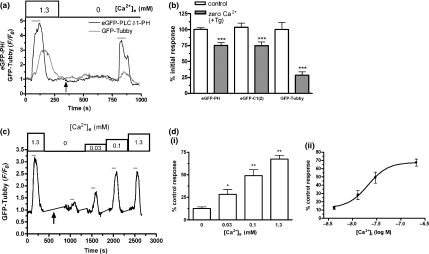
Determining the Ca^2+^-sensitivity of PLC activity using GFP-labelled biosensors. (a) Representative traces demonstrating eGFP-PH (black line) and GFP-Tubby (grey line) responses to MCh (100 μM; grey horizontal bars) in SH-SY5Y cells in Krebs–Henseleit buffer (KHB) containing 1.3 mM Ca^2+^, and under Ca^2+^-free (0 Ca^2+^) conditions and addition of thapsigargin (5 μM; black arrow) to deplete intracellular Ca^2+^ stores. Data were expressed as a ratio change in cytosolic fluorescence emission (*F*) relative to the initial basal fluorescence (*F*_0_). (b) Cumulative data representing eGFP-PH, GFP-Tubby and eGFP-C1(2) translocation in response to MCh (100 μM) in KHB containing 1.3 mM Ca^2+^ (control) and in nominally Ca^2+^-free following the addition of thapsigargin (5 μM) (0 Ca^2+^ + Tg). Data were presented as a mean percent of an initial control response achieved in the presence of 1.3 mM Ca^2+^. Differences between control and Ca^2+^-free responses were determined by one way anova and Bonferroni’s *post hoc* test (***p<0.001). (c) Representative trace illustrating GFP-Tubby responses to MCh (100 μM; grey horizontal bars) in SH-SY5Y cells in KHB containing 1.3 mM Ca^2+^ and in 0 (nominally Ca^2+^-free), 0.03, 0.1 or 1.3 mM Ca^2+^ following addition of thapsigargin (5 μM; black arrow). Data were expressed as a ratio change in cytosolic fluorescence emission (*F*) relative to the initial basal fluorescence (*F*_0_). [d(i)] Cumulative data representing GFP-Tubby translocation in SH-SY5Y cells following thapsigargin (5 μM) treatment in nominally free extracellular Ca^2+^ and subsequent stimulation with MCh (100 μM) in the presence of KHB containing 0, 0.03, 0.1 and 1.3 mM Ca^2+^. Responses were normalized to the initial control response achieved in 1.3 mM Ca^2+^ prior to thapsigargin treatment and are expressed as mean percent of control response. Differences between responses in nominally Ca^2+^-free KHB (0 Ca^2+^) and those in the presence of increasing concentrations of extracellular Ca^2+^ were determined by one-way anova and Dunnett’s *post hoc* test (*p<0.05; **p<0.01). [d(ii)] Concentration–response curve representing GFP-Tubby responses to MCh (100 μM) as a function of the intracellular Ca^2+^ concentration (determined from Fluo-4 emissions) after thapsigargin (5 μM) treatment and the establishment of a steady state level of [Ca^2+^]_i_. Responses were normalized to the initial control response achieved in 1.3 mM Ca^2+^ prior to thapsigargin treatment and are expressed as mean percent of this control response. Where appropriate, data were expressed as mean ± SEM for four or more cells from at least three separate coverslips.

To investigate the Ca^2+^-dependence of MCh-stimulated PLC activity in SH-SY5Y cells, we examined the effects of manipulating intracellular Ca^2+^ levels on the magnitude of translocations of eGFP-PH, eGFP-C1(2) and GFP-Tubby in response to MCh. Removal of extracellular Ca^2+^ and depletion of intracellular Ca^2+^ stores with thapsigargin (5 μM) completely abolished Ca^2+^ responses, measured using Fluo-4 AM, to MCh (100 μM) (data not shown). MCh-mediated GFP-Tubby translocation was substantially reduced under Ca^2+^-depletion conditions, while eGFP-PH translocation in response to MCh was significantly, but much less markedly attenuated (Fig. 4a). Across a number of experiments, similar profiles were observed with both eGFP-PH and eGFP-C1(2), where depletion of intracellular Ca^2+^ stores in the absence of extracellular Ca^2+^ attenuated the response observed with both probes by ∼25% (Fig 4b). In contrast, under similar conditions the magnitude of GFP-Tubby translocation in response to MCh (100 μM) was reduced by around 70% (Fig. 4b). The greater sensitivity of GFP-Tubby translocation to Ca^2+^ availability was exploited in experiments to assess the concentration range over which M_3_ mACh receptor-stimulated PLC activity is sensitive to [Ca^2+^]_i_. Following an initial control response to MCh (100 μM), cells were perfused with Ca^2+^-free buffer and treated with thapsigargin (5 μM) to deplete intracellular Ca^2+^ stores. A second MCh, response was obtained and on washout, extracellular [Ca^2+^] was subsequently raised to 0.03, 0.1 and finally 1.3 mM, with further responses to MCh (100 μM) assessed by GFP-Tubby translocation under each condition (Fig. 4c). Raising [Ca^2+^]_e_ from 0 to just 0.03 mM was sufficient significantly to enhance the GFP-Tubby response to MCh, while approximately two-thirds of the response was recovered in the presence of 1.3 mM [Ca^2+^]_e_ (Fig. 4d). Thapsigargin treatment and subsequent changes in [Ca^2+^]_e_ alone had no direct effect on GFP-Tubby localization.

To equate the changes in extracellular [Ca^2+^] with intracellular Ca^2+^ levels (and therefore the [Ca^2+^] experienced by PLC), Fluo-4 AM-loaded SH-SY5Y cells were subjected to the protocol described above (without the MCh treatments). A typical trace is shown in Fig. S2. Estimates of the intracellular Ca^2+^ concentration under these conditions were obtained using the method described by Maravall *et al.* (2000) for estimating intracellular Ca^2+^ concentrations from single excitation Ca^2+^-indicators such as Fluo-4. Mean resting [Ca^2+^]_i_ levels in SH-SY5Y cells were ∼53 nM, while following treatment with thapsigargin in the presence of Ca^2+^-free buffer, intracellular Ca^2+^ levels fell to as low as 4 nM. Mean [Ca^2+^]_i_ following ‘add-back’ of different levels of extracellular Ca^2+^ were determined and plotted against % control response, to generate an estimated mean [Ca^2+^]_i_ concentration–response curve for MCh-stimulated GFP-Tubby translocation [Fig. 4d(ii)]. From this curve, the [Ca^2+^]_i_ required for half-maximal rescue of MCh-stimulated PLC activation can be estimated to be around 20 nM, indicating that low levels of intracellular Ca^2+^ are sufficient to facilitate the activation of PLC by Gα_q/11_-coupled receptor systems in SH-SY5Y neuroblastoma cells. These data were consistent with the observation that IP_3_ 3-kinase over-expression had no effect on GFP-Tubby/eGFP-C1(2) responses (Fig. 2), as the residual Ca^2+^ responses to 100 μM MCh in cells over-expressing the 3-kinase (see Fig. 2b) would be sufficient to permit maximal agonist-mediated PLC activity.

### Visualizing PIP_2_ in cultured rat hippocampal neurons using GFP-Tubby

**Fig. 5:**
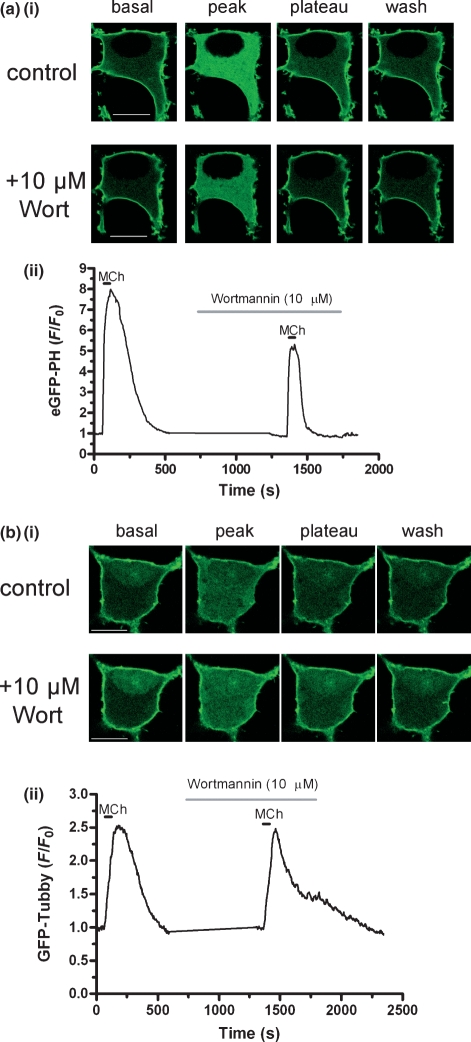
The effect of PI4-kinase inhibition on translocation of GFP-labelled biosensors in response to MCh in cultured neonatal rat hippocampal neurons. Representative images (i) and traces (ii) of eGFP-PH (a) and GFP-Tubby (b) translocation, in response to MCh (1 mM; black horizontal bar), in cultured rat hippocampal neurons (15 DIV) in the absence and presence of pre-incubated wortmannin (10 μM; grey horizontal bar). Data were expressed as a ratio change in cytosolic fluorescence emission (*F*) relative to the initial basal fluorescence (*F*_0_) and are representative of at least 11 cells from three or more coverslips. Scale bars, 10 μm.

We have previously used the eGFP-PH biosensor to investigate a variety of aspects of PLC signalling in cultured hippocampal neurons (see Nahorski *et al.* 2003) and have provided evidence that in this system, eGFP-PH translocation largely reflects changes in IP_3_ levels (Nash *et al.* 2004). Indeed, stimulation (with 1 mM MCh) of the endogenous mACh receptor population expressed in rat hippocampal neurons, cultured for 15 DIV, elicited a robust translocation of eGFP-PH from plasma membrane to cytosol. Following agonist washout, eGFP-PH re-localized to the plasma membrane [Fig. 5a(i)]. In cells pre-treated with either 1 μM (data not shown) or 10 μM Wort (Fig. 5a), similar temporal response profiles were observed, with no significant effect on either the peak height or plateau level of response relative to control cells (Table S3). These data were consistent with eGFP-PH translocation reporting changes in intracellular IP_3_ rather than depletion of plasma membrane PIP_2_ in cultured hippocampal neurons (Nash *et al.* 2004).

However, until now, the ability selectively to image PIP_2_ in neurons has been lacking. We therefore expressed GFP-Tubby in cultured hippocampal neurons, where it exhibited a predominantly plasma membrane localization at rest and a substantial membrane-to-cytosol translocation on activation of the PLC pathway (Fig. 5b), similar to that observed in other cell backgrounds (Santagata *et al.* 2001 and this study). In control cells (and in those pre-treated with 1 μM Wort), GFP-Tubby re-localized to the plasma membrane on agonist washout, but in the presence of 10 μM Wort, the re-association of the probe to the plasma membrane was significantly slowed, with a plateau phase of response (∼35% of peak response) maintained until washout of Wort (Fig. S5b and Table S3). However, no significant differences in peak height were observed between control cells and those pre-incubated with either 1 or 10 μM Wort (Table S3).

Although the above data were qualitatively consistent with our findings in SH-SY5Y cells (see Table S2), the effect of Wort (10 μM) on GFP-Tubby translocation in hippocampal neurons in response to MCh was modest (Fig. S5b and Table S3). We therefore investigated whether exposure to agonist (MCh) for longer periods would further deplete plasma membrane PIP_2_ and therefore reveal a more pronounced effect of PI4-kinase inhibition on the re-localization of GFP-Tubby following agonist washout. In cells exposed to MCh (1 mM) for 3 min, GFP-Tubby cytosolic fluorescence decreased to baseline following agonist washout in control cells, while in cells pre-incubated with Wort (10 μM), the response was maintained at a plateau level until Wort was washed out (see Fig. S3). Over a number of experiments, plateau values (derived as % of peak response 240 s after peak) in control cells were only 6 ± 3% (*n* = 5), while those in cells pre-treated with Wort (10 μM) were significantly higher [61 ± 13% (*n* = 6); *p*<0.01; anova and Bonferroni’s *post hoc* test]. In contrast, GFP-Tubby responses in cells pre-treated with 1 μM Wort exhibited plateau values not significantly different to control [20 ± 8% (*n* = 5)]. Overall, these data suggest that, as in SH-SY5Y neuroblastoma cells, the translocation of GFP-Tubby reports dynamic changes in plasma membrane PIP_2_ in hippocampal neurons.

## Discussion

We have used GFP-labelled biosensors to study, in real-time, dynamic changes in the levels of PIP_2_ and the products of PLC-mediated PIP_2_ hydrolysis, in intact living cells. Use of eGFP-PH, eGFP-C1(2) and GFP-Tubby has also allowed us to clarify the specificity of these probes and to address questions regarding the regulation of PLC activity in human neuroblastoma cells and primary neurons.

### eGFP-PH detects IP_3_

A key finding of the present investigation is that translocation of eGFP-PH in response to mACh receptor stimulation in SH-SY5Y cells was dependent on changes in the cytosolic levels of IP_3_. Over-expression of IP_3_ 3-kinase, to attenuate the IP_3_ response to MCh, substantially reduced eGFP-PH translocation, indicating a predominant role for IP_3_ in facilitating translocation of this biosensor. This is in agreement with the findings of previous studies in a variety of cell backgrounds, including Madin-Darby canine kidney cells (Hirose *et al.* 1999), Purkinje neurons (Okubo *et al.* 2001), Chinese hamster ovary cells (Nash *et al.* 2002) and hippocampal neurons (Nash *et al.* 2004). The over-expression of enzymes involved in the metabolism of IP_3_ to investigate the role of changes in IP_3_ levels in eGFP-PH translocation has been criticized, as this intervention also removes the IP_3_-mediated Ca^2+^ release which can potentiate PLC activity in some systems (Horowitz *et al.* 2005; Varnai and Balla 2006; Quinn *et al.* 2008). However, in this study, IP_3_ 3-kinase over-expression had no effect on DAG production [determined using the DAG sensor eGFP-C1(2)], indicating that PLC activity was not compromised by IP_3_ 3-kinase over-expression. This strongly suggests that the diminished eGFP-PH responses observed under these conditions reflect the IP_3_-dependence of the translocation. However, it should be noted that if DAG metabolism is also Ca^2+^-dependent (e.g. DAG kinase activity may be regulated by Ca^2+^; Yamada *et al.* 1997), IP_3_ 3-kinase over-expression could decrease DAG metabolism, masking any decrease in DAG production resulting from reduced PLC activity.

Other reports have suggested the predominant role of PIP_2_ in mediating eGFP-PH translocation, so how can these divergent findings be reconciled? van der Wal *et al.* (2001) reported that inhibition of PIP_2_ re-synthesis with the PI4-kinase inhibitor phenylarsine oxide inhibited the re-localization of eGFP-PH to the plasma membrane. Indeed, our own experiments using inhibitors of PI4-kinase (Wort and LY294002) suggest that PIP_2_ re-synthesis is essential for the *full* re-association of eGFP-PH with the plasma membrane. However, as the eGFP-PH domain of PLCδ1 associates with PIP_2_ at the membrane, inhibition of PIP_2_ re-synthesis would be expected to impair the re-localization of the probe. Similarly, eGFP-PH translocation may be initiated by a rapid and profound depletion of PIP_2_ following the activation of a PIP_2_ 5-phosphatase, in the absence of a rise in [IP_3_] (Suh *et al.* 2006; Quinn *et al.* 2008). However, our data and that of others (see earlier) indicate that the rapid initial translocation of the probe away from the membrane in response to agonist stimulation is more likely to be driven by the increase in IP_3_. This is further supported by our own calculations (see Fig. S4) and by the modelling simulations (based on empirically derived parameters) of Xu *et al.* (2003), who found that although changes in either PIP_2_ or IP_3_ were capable of translocating eGFP-PH, experimental data were better modelled by simulations in which IP_3_ levels changed, while the PIP_2_ concentration remained constant.

The observation that only very high (≥ 10 μM) levels of IP_3_ are able to initiate the translocation of eGFP-PH (van der Wal *et al.* 2001) provides another argument against the predominant role of IP_3_ in driving the translocation, as such levels of IP_3_ might not be achieved physiologically, even in response to maximal agonist stimulation. However, the higher IP_3_ concentrations are only required to translocate eGFP-PH because under those experimental conditions, [PIP_2_] remained at a constant (high) level, competing with IP_3_ for binding to eGFP-PH. In addition, Hirose *et al.* (1999) found that 1 μM IP_3_ was sufficient to induce a marked eGFP-PH translocation in Madin-Darby canine kidney cells. Xu *et al.* (2003) addressed these contrasting findings with further modelling work and found that the eGFP-PH response to a 1 μM bolus of IP_3_ was predicted to be highly sensitive to eGFP-PH expression levels, over only a 10-fold range, suggesting that such conflicting results might be because of differences in the expression levels of eGFP-PH biosensor. The relative contribution of PIP_2_ to the translocation of eGFP-PH may be greater in some cell types than others and may be influenced by a variety of experimental conditions (Varnai and Balla 2006), but it is clear that eGFP-PH does not provide a selective means of measuring dynamic changes in cellular PIP_2_.

### GFP-Tubby detects PIP_2_

Tubby protein has previously been shown to bind with high affinity and selectivity to PIP_2_ in live cells, leading to the plasma membrane localization of a GFP-labelled form of the protein (Santagata *et al.* 2001). When expressed in SH-SY5Y cells, GFP-Tubby translocated in an agonist concentration-dependent manner in response to mACh receptor stimulation and this translocation was independent of changes in intracellular IP_3_, in agreement with a recent study using a modified version of the Tubby domain (Quinn *et al.* 2008). Crucially, in the presence of PI4-kinase inhibition, peak GFP-Tubby responses were significantly enhanced and were maintained beyond agonist washout. It was only on the removal of PI4-kinase inhibition (allowing PIP_2_ re-synthesis to resume) that GFP-Tubby was able to re-associate with the plasma membrane. Although the inhibitors used in these experiments (Wort and LY294002) are not specific for PI4-kinase, the consistency between the data obtained with each inhibitor (and the lack of effect of a lower, PI3-kinase-inhibiting concentration of Wort) strongly supports the notion that PI4-kinase activity is crucial for the re-association of GFP-Tubby to the plasma membrane. We therefore believe that GFP-Tubby translocation provides a selective means of visualizing changes in cellular PIP_2_, without the confounding influence of changes in IP_3_ concentration.

### PLC signalling in living cells

The availability of selective fluorescent biosensors for IP_3_, DAG and PIP_2_, allowed us to perform a comprehensive investigation of PLC signalling in SH-SY5Y cells. Comparison of the kinetics and potencies of translocation for each biosensor highlighted some interesting differences. First, in response to the same agonist stimulus, GFP-Tubby translocated substantially more slowly than eGFP-PH, which in turn was marginally slower than eGFP-C1(2). Earlier biochemical measurements in SH-SY5Y cells indicate that the decrease in PIP_2_ observed on MCh stimulation reaches a peak at around 60 s (Willars *et al.* 1998), consistent with the time-course of GFP-Tubby translocation (*t*_10–90_ = 59 s), while IP_3_ mass responses peak more quickly (∼10–15 s; Willars *et al.* 1998). However, as GFP-Tubby consists of the full-length Tubby protein labelled with GFP, it is substantially larger (molecular mass ∼83 kDa) than either eGFP-PH (∼41 kDa) or eGFP-C1(2) (∼34 kDa). This raises the possibility that differences in the kinetics of the translocation of the three biosensors may reflect differences in their respective rates of diffusion. The second notable difference was the approximately fivefold lower potency of MCh for inducing translocation of GFP-Tubby, relative to eGFP-PH. If both probes are reporting the activation of the PLC pathway, it might be anticipated that a given agonist should elicit translocation of each biosensor with equal potency. However, while both eGFP-PH and GFP-Tubby localize to the plasma membrane under basal conditions, translocation of the former is primarily triggered by an increase in IP_3_, whereas that of GFP-Tubby relies purely on hydrolysis of PIP_2_. As the concentration of PIP_2_ is high at rest (360 pmol/mg protein in SH-SY5Y cells; Willars *et al.* 1998) it is possible that GFP-Tubby migration (between PIP_2_ molecules) competes with translocation of the probe into the cytosol, causing a rightward shift in the concentration–response curve relative to that reported by eGFP-PH (which, unlike GFP-Tubby, benefits from the ‘pull’ of the elevated cytosolic IP_3_ concentration). In addition, the translocation of GFP-Tubby reports the *net* change in concentration of its target molecule and will therefore reflect the influence of both synthesis and metabolism of this target. Given that PIP_2_ synthesis may be stimulated alongside PLC activation in some cases (e.g. Xu *et al.* 2003; Winks *et al.* 2005), it is conceivable that changes in PIP_2_ and IP_3_ levels might become uncoupled because of differences in their relative rates of synthesis/metabolism, despite the equivalent level of PLC activity resulting from mACh receptor activation. Although Gamper *et al.* (2004) found that M_1_ mACh receptor activation did not stimulate PIP_2_ synthesis (while bradykinin B_2_ receptor stimulation did) in rat superior cervical ganglion cells, this was considered to be because of the absence of a Ca^2+^ response to M_1_ mACh receptor activation in ganglion cells. As we observed robust Ca^2+^ responses to MCh in SH-SY5Y cells, it is possible that in this system mACh receptor activation could stimulate PIP_2_ synthesis and this requires further investigation.

### Agonist-stimulated PLC activity is highly sensitive to [Ca^2+^]_i_

Agonist-stimulated PLC activity has been known for some time to be dependent on intracellular Ca^2+^ (Eberhard and Holz 1988) and the availability of fluorescently labelled biosensors for the substrate (PIP_2_) and both products (DAG and IP_3_) of PLC allowed us directly to visualize the Ca^2+^-sensitivity of PLC activity in neuroblastoma cells. Although direct activation of various PLC isoenzymes by Ca^2+^ in the micromolar range has been demonstrated (Allen *et al.* 1997; Hwang *et al.* 2005), we did not observe translocation of any of the three biosensors under investigation following treatment of SH-SY5Y cells with ionomycin (3 μM), even though this caused a substantial rise in intracellular Ca^2+^ (data not shown). In contrast, Varnai and Balla (1998) found that ionomycin treatment translocated eGFP-PH in NIH-3T3 cells, perhaps reflecting cell background-dependent differences in PLC sensitivity to Ca^2+^. However, removal of extracellular Ca^2+^ and depletion of intracellular Ca^2+^ stores (reducing intracellular Ca^2+^ concentrations to the low nanomolar range) significantly attenuated MCh-stimulated translocation of eGFP-PH, GFP-Tubby and eGFP-C1(2). Although both eGFP-C1(2) and eGFP-PH responses were reduced by a similar amount following Ca^2+^ depletion/removal, GFP-Tubby responses were more substantially attenuated. This suggests that, in addition to inhibiting agonist-mediated PLC activity, lowering intracellular Ca^2+^ may have additional effects on the metabolism/synthesis of one or more of the signalling intermediates (PIP_2_/DAG/IP_3_) being detected by these biosensors. It is interesting to note that Quinn *et al.* (2008) found that translocation of R332H-Tubby (248–505)-yellow fluorescent protein was reduced under Ca^2+^-buffered conditions, while eGFP-PH was unaffected, providing further evidence that Tubby is more sensitive to the effects of Ca^2+^ on PLC activity.

We exploited the greater responsiveness of GFP-Tubby responses to changes in intracellular Ca^2+^ to demonstrate that agonist-mediated PLC activity appears to be highly sensitive to Ca^2+^ in SH-SY5Y cells. By adding Ca^2+^ back to the bath solution in a graded manner, we were able to demonstrate that maximal recovery was obtained by raising intracellular Ca^2+^ to between 100 and 200 nM, indicating the exquisite sensitivity of agonist-mediated PLC activity around the physiological resting Ca^2+^ concentration. Although this suggests a greater sensitivity than reported for carbachol-mediated stimulation of PLC-β1 in re-constituted vesicles (Biddlecome *et al.* 1996), our findings are in agreement with a number of earlier studies in a variety of other cell systems (Renard *et al.* 1987; del Rio *et al.* 1994; Young *et al.* 2003; Horowitz *et al.* 2005), indicating that agonist-stimulated PLC activity may be more sensitive to Ca^2+^ in its native environment. Our data also concur with earlier findings in SH-SY5Y cells, where carbachol-stimulated PLC activity (measured biochemically) was highly dependent on the free Ca^2+^ concentration in the range of 20–100 nM (Wojcikiewicz *et al.* 1994).

Although we have not identified the PLC isoenzyme(s) mediating agonist-stimulated PIP_2_ hydrolysis in the systems investigated in this study, PLC-β1 is the predominant isoform activated by mACh receptors in SH-SY5Y cells (Sorensen *et al.* 1998) and it is also highly expressed in the hippocampus (Rebecchi and Pentyala 2000). However, members of the novel PLC-η family are localized to neuronal cells and are also highly sensitive to Ca^2+^ (Cockcroft 2006). A direct physiological role for PLC-η enzymes in linking intracellular Ca^2+^ levels to PLC activity has yet to be established, but this remains an intriguing area for further study.

### PIP_2_ and IP_3_ dynamics in primary neurons

Finally, we have shown that GFP-Tubby can be used to visualize dynamic changes in PIP_2_ in mature cultured hippocampal neurons. Stimulation of the endogenous mACh receptor population expressed in these cultures elicited a concentration-dependent translocation of GFP-Tubby. Inhibition of PI4-kinase activity with Wort led to a component of the GFP-Tubby response being maintained beyond agonist washout, although a longer agonist exposure (3 min) generated a more robust plateau in the response. This suggests that GFP-Tubby translocation reflects changes in plasma membrane PIP_2_, but that a more sustained stimulus may be required in this system sufficiently to deplete PIP_2_ (even in the presence of Wort) to prevent the probe re-associating with the membrane. In contrast, eGFP-PH translocation in response to MCh was unaffected by Wort, consistent with the probe detecting changes in IP_3_ levels in hippocampal neurons as reported by us earlier (Nash *et al.* 2004).

In summary, we have demonstrated the utility of GFP-Tubby as a fluorescent ‘biosensor’ for PIP_2_ and, in conjunction with well-characterized fluorescent probes for IP_3_ (eGFP-PH) and DAG [eGFP-C1(2)], have provided a comprehensive analysis of the Ca^2+^-sensitivity of PLC activity in live cells. Depolarization-induced Ca^2+^ entry has been shown to potentiate G protein-coupled receptor-driven PLC activity in neurons, with PLC-β1 acting as the ‘coincidence detector’ (Hashimotodani *et al.* 2005). In addition, we have previously reported that G_q/11_-coupled receptor-mediated IP_3_ responses in single hippocampal neurons may be enhanced by coincident glutamate-mediated synaptic activity and α-amino-3-hydroxy-5-methyl-4-isoxazole propionic acid receptor-activated Ca^2+^ entry (Nash *et al.* 2004, Willets *et al.* 2007). Given the important and distinct roles in neuronal physiology fulfilled by PLC substrate and products, the potential for this effector to provide the focal point for the integration of metabotropic and ionotropic signalling in neurons clearly merits further investigation. We anticipate that the use of selective biosensors for PIP_2_, IP_3_ and DAG will provide crucial new insights into the regulation of PLC activity at the single cell and subcellular level, facilitating the further study of this ubiquitous signalling pathway.

## Abbreviations used

DAG: diacylglycerol
DIV: days *in vitro*
eGFP: enhanced GFP
eGFP-C1(2): eGFP-protein kinase C-γ-C1_2_
eGFP-PH: eGFP-PH-PLCδ1
GFP: green fluorescent protein
IP_3_: inositol 1,4,5-trisphosphate
mACh: muscarinic acetylcholine
MCh: methacholine
PI3-kinase: phosphoinositide 3-kinase
PIP_2_: phosphatidylinositol 4,5-bisphosphate
PLC: phospholipase C
Wort: wortmannin

## Supporting information

**Fig. S1** Concentration–response curves for eGFP-PLCδ1 PH (a), GFP-Tubby (b) or eGFP-PKCγ C1(2) (c) translocation in response to MCh in SH-SY5Y cells.

**Fig. S2** Fluo-4 emission trace, illustrating relative changes in [Ca^2+^]_i_ in a representative SH-SY5Y cell in response to changes in [Ca^2+^]_e_ and thapsigargin treatment.

**Fig. S3** Longer agonist treatment enhances PIP_2_ depletion in the presence of PI4-kinase inhibition in cultured neonatal rat hippocampal neurons.

**Fig. S4** Simulation of the relationship between PIP_2_ concentration and eGFP-PH localization in SH-SY5Y cells.

**Table S1** Concentration-dependence and kinetics of GFPlabelled biosensor translocations in response to MCh in SH-SY5Y cells.

**Table S2** eGFP-PH and GFP-Tubby translocation in SH-SY5Y cells in response to MCh (1 mM), in the absence (control) and presence of wortmannin (Wort; 1 or 10 μM) or LY294002 (LY; 100 μM).

**Table S3** eGFP-PH and GFP-Tubby translocation in cultured neonatal rat hippocampal neurons in response to MCh (1 mM), in the absence (control) and presence of wortmannin (Wort; 1 or 10 μM).
