# Supplementary material for: Temporal profiling of changes in phosphatidylinositol 4,5-bisphosphate, inositol 1,4,5-trisphosphate and diacylglycerol allows comprehensive analysis of phospholipase C-initiated signalling in single neurons1
Source: J Neurochem. 2008 Nov;107(3):602–15. doi: 10.1111/j.1471-4159.2008.05587.x (PMC2779467; doi:10.1111/j.1471-4159.2008.05587.x)
Supplement: Supplementary file 1 [file jnc0107-0602-SD1.pdf]

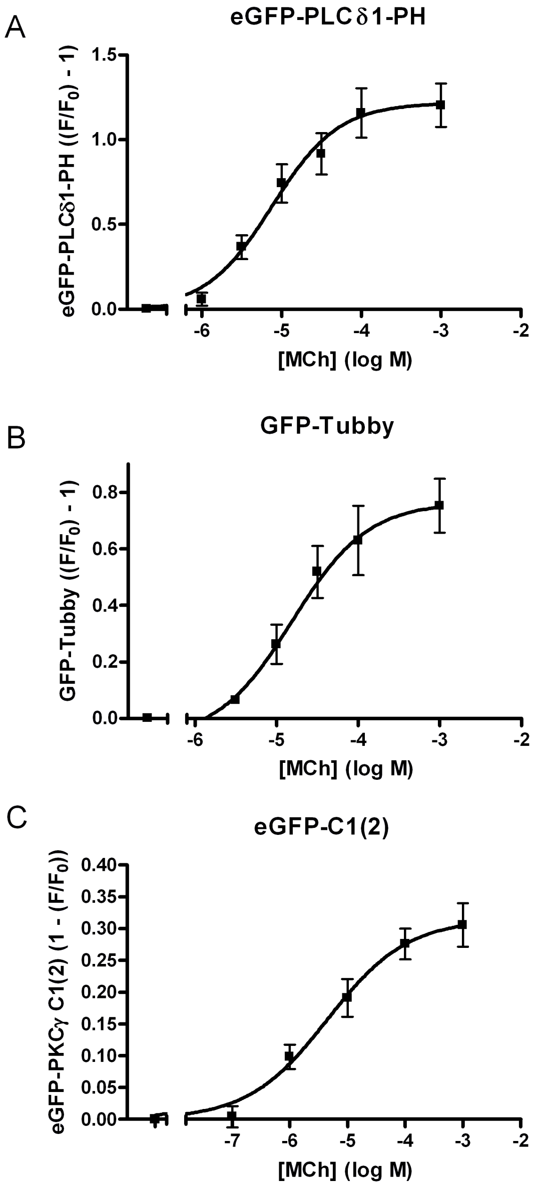

Supplementary Fig. 1

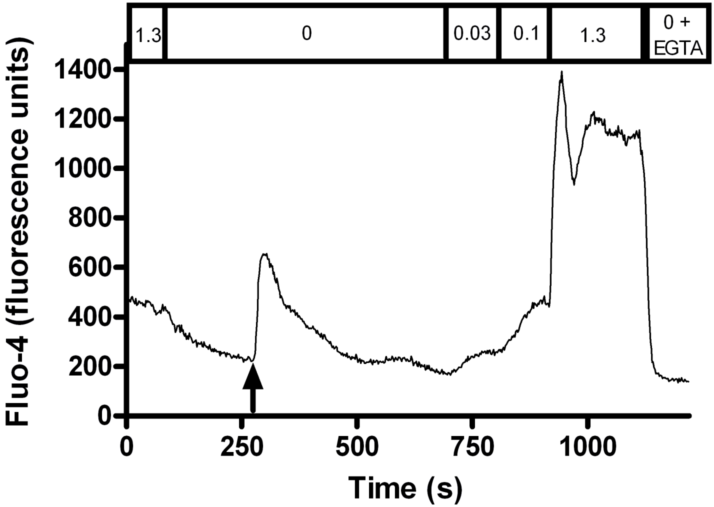

Supplementary Fig. 2

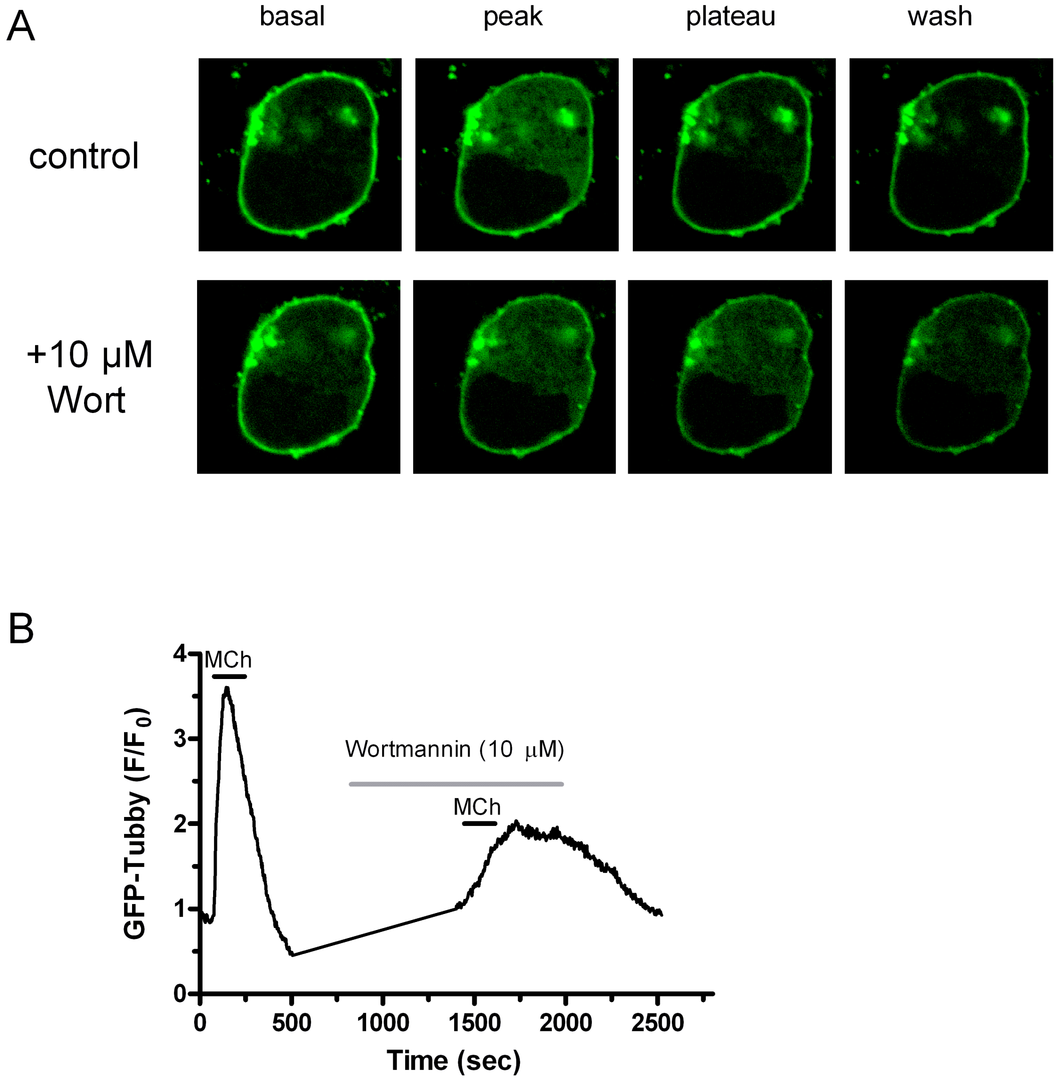

Supplementary Figure 3

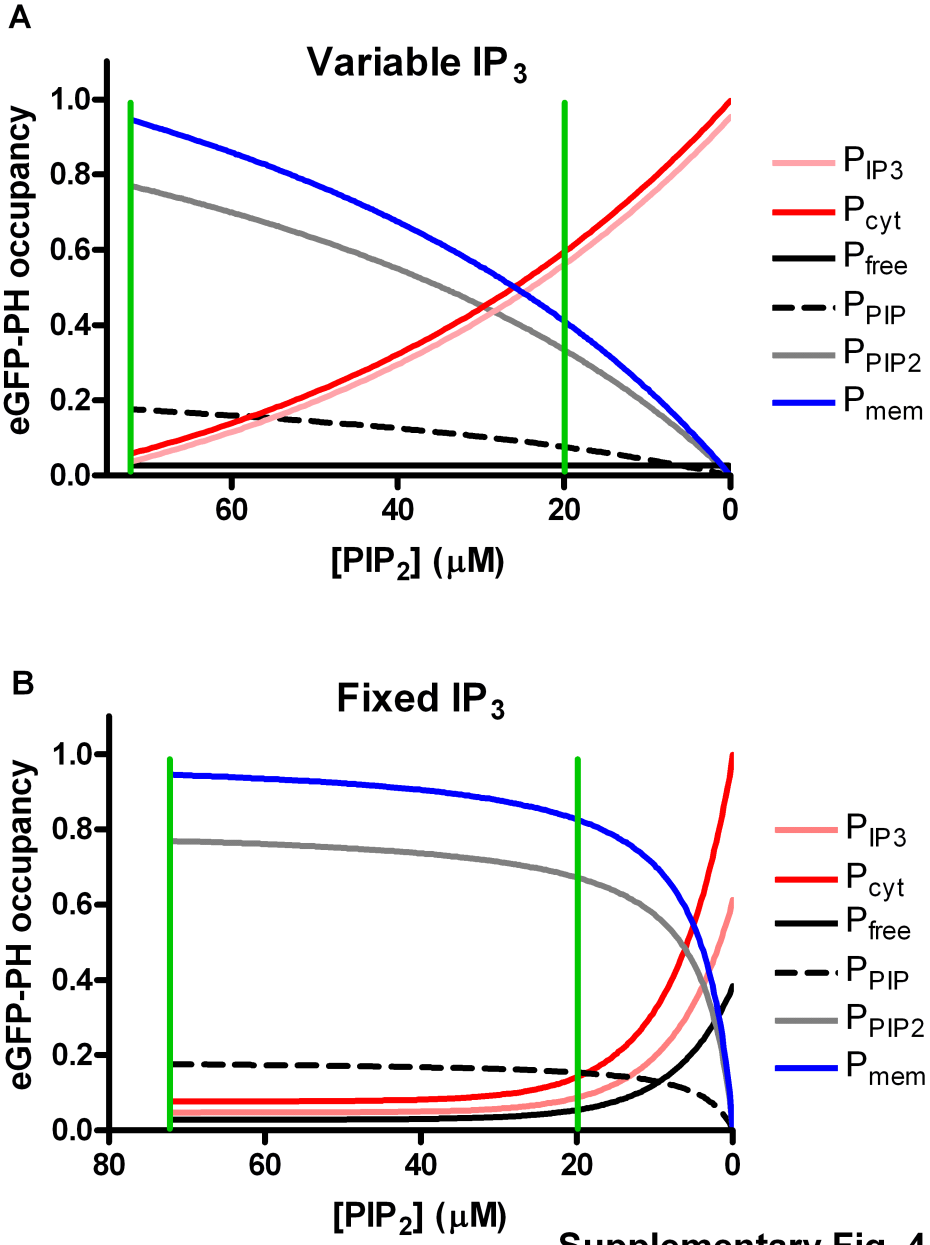

Supplementary Fig. 4

Supplementary Table 1

Concentration-dependence and kinetics of GFP-labeled biosensor translocations in response to MCh in SH-SY5Y cells.

	**eGFP-PH**	**GFP-Tubby**	**eGFP-C1(2)**
**pEC_{50} (M)**	5.19 ± 0.11 (9)	4.53 ± 0.21 (5)*	4.89 ± 0.23 (6)
**t_{10-90} (sec)**	39.2 ± 2.6 (35)	58.8 ± 3.0 (30)***	30.8 ± 1.7 (48)††† ‡

Mean pEC_{50} (negative log of the agonist concentration required to produce 50% of the maximal response) and t_{10-90} (time (in sec) between 10 and 90% of peak response to MCh (100 μM)) estimates for eGFP-PH, GFP-Tubby and eGFP-C1(2) translocation in SH-SY5Y cells in response to MCh. Differences between pEC_{50} or t_{10-90} estimates were determined by one-way ANOVA and Bonferroni's *post-hoc* test, with * denoting differences between eGFP-PH and GFP-Tubby ($^{*}P<0.05$; $^{***}P<0.001$), † denoting differences between eGFP-C1(2) and GFP-Tubby ($^{†††}P<0.001$) and ‡ denoting differences between eGFP-C1(2) and eGFP-PH ($^{‡}P<0.05$).

Supplementary Table 2

eGFP-PH and GFP-Tubby translocation in SH-SY5Y cells in response to MCh (1 mM), in the absence (control) and presence of wortmannin (Wort; 1 or 10 μM) or LY294002 (LY; 100 μM).

	**control**	**+ 1 μM Wort**	**control**	**+ 10 μM Wort**	**control**	**+ 100 μM LY**
**eGFP-PH**						
peak	3.38 ± 0.43 (8)	3.60 ± 0.40 (8)	3.77 ± 0.25 (14)	2.97 ± 0.17 (14)*	3.69 ± 0.33 (6)	2.46 ± 0.24 (6)*
plateau	-1.2 ± 4.9 (8)	4.7 ± 1.6 (8)	0.9 ± 2.6 (15)	43.5 ± 6.4 (15)**	0.4 ± 3.1 (6)	32.9 ± 6.7 (6)**
**GFP-Tubby**						
peak	2.33 ± 0.27 (9)	2.69 ± 0.32 (9)	2.97 ± 0.58 (9)	4.93 ± 0.67 (9)*	1.98 ± 0.29 (7)	2.33 ± 0.22 (7)
plateau	-2.1 ± 2.8 (8)	-6.0 ± 3.4 (8)	2.4 ± 4.9 (9)	78.9 ± 6.3 (10)***	1.3 ± 1.9 (7)	62.0 ± 4.2 (7)***

Data are expressed as means ± s.e.m. (number of cells indicated in parentheses) for fold changes in cytosolic fluorescence over basal (F/F_0) (for "peak" values) or percent of peak responses remaining 240 sec after peak (for "plateau" values). Differences between control and inhibitor-treated cells were determined by Student's *t*-test ($^{*}P<0.05$; $^{**}P<0.01$; $^{***}P<0.001$).

Supplementary Table 3

eGFP-PH and GFP-Tubby translocation in cultured neonatal rat hippocampal neurons in response to MCh (1 mM), in the absence (control) and presence of wortmannin (Wort; 1 or 10 μM).

	**Control**	**+ 1 μM Wort**	**+ 10 μM Wort**
**eGFP-PH**			
peak	3.33 ± 0.51 (26)	2.69 ± 0.60 (12)	2.78 ± 0.52 (11)
plateau	5.3 ± 2.8 (26)	5.4 ± 3.9 (12)	6.2 ± 3.2 (11)
**GFP-Tubby**			
peak	1.47 ± 0.08 (23)	1.29 ± 0.05 (7)	1.54 ± 0.21 (11)
plateau	1.4 ± 4.5 (23)	4.7 ± 3.6 (6)	35.8 ± 7.7 (11)***

Data are expressed as means ± s.e.m. (number of cells indicated in parentheses) for fold changes in cytosolic fluorescence over basal (F/F_0) (for "peak" values) or percent of peak responses remaining 240 sec after peak (for "plateau" values). Differences between control and inhibitor-treated cells were determined by one-way ANOVA and Bonferroni's *post-hoc* test ($^{***}P<0.001$).
